# Seawater Biodegradable Poly(butylene succinate-*co*-adipate)—Wheat Bran Biocomposites

**DOI:** 10.3390/ma16072593

**Published:** 2023-03-24

**Authors:** Giovanna Strangis, Damiano Rossi, Patrizia Cinelli, Maurizia Seggiani

**Affiliations:** Department of Civil and Industrial Engineering, University of Pisa, Largo Lucio Lazzarino 1, 56122 Pisa, Italy

**Keywords:** poly(butylene succinate-*co*-adipate) (PBSA), biodegradable bran seawater, biocomposites, filler

## Abstract

The present work focused on the development and characterization of biocomposites based on a fully bio-based polyester, poly(butylene succinate-*co*-butylene adipate) (PBSA), and wheat bran derived by flour milling. PBSA-bran composites containing 5, 10, 15, and 20 wt.% of wheat bran were produced via melt extrusion and processed by injection molding. Their thermal, rheological, morphological, and tensile properties were investigated. In addition, a biodegradation test in a natural marine environment was conducted on composite dog-bones to assess the capacity of the used filler to increase the PBSA biodegradation rate. The composites maintained similar melt processability and mechanical properties to virgin PBSA with up to 15 wt.% bran content. This result was also supported by morphological investigation, which showed good filler dispersion within the polymer matrix at low-mid bran content, whereas poor polymer-filler dispersion occurred at higher concentrations. Furthermore, the biodegradation tests showed bran’s capacity to improve the PBSA biodegradation rate, probably due to the hygroscopic bran swelling, which induced the fragmentation of the dog-bone with a consequent increase in the polymeric matrix–seawater interfacial area, accelerating the degradation mechanisms. These results encourage the use of wheat bran, an abundant and low-cost agri-food by-product, as a filler in PBSA-based composites to develop products with good processability, mechanical properties, and controlled biodegradability in marine environments.

## 1. Introduction

The accumulation of non-biodegradable plastic waste is a pressing environmental issue that is receiving great attention from government authorities and the research world. The global production of petroleum-based plastics is still increasing due to their strength, lightweight, adaptability, and low cost [1,2,3]. Fuel-based plastics cover a wide range of applications in the domestic, medical, and industrial fields, becoming indispensable in our lives. It was estimated that 390.7 million tons of plastics were produced globally in 2021, of which roughly 23% are still landfilled or inappropriately disposed of into the natural environment, especially in water bodies or seas [4]. Annual production volumes are expected to continue rising in the following decades to approximately 590 million tons by 2050. Every year, about 13 million tons of plastics reach the oceans [5], and the accumulation in the seas causes harmful impacts on wildlife and human health [6,7]. Plastic recycling is currently the most widely adopted technique to minimize these impacts as it reduces carbon emissions due to the lower volumes of plastic produced and finally disposed. On the other hand, chemical and mechanical recycling may cause a reduction in the properties of materials that compromise their reuse [8,9,10]. In other words, recycling strategies are not unlimited and can only be pursued for a defined amount of time up until reaching the end of the product life cycle, where the unavoidable material deterioration and unusability lead to the consequent need for product incineration or landfilling [11,12]. For this reason, to address the problem of petrochemical-based plastic disposal, the research interest has moved toward the direction of biodegradable polymers as alternative materials to solve plastic waste issues. The strategy to focus on this class of polymers is a remarkable challenge that requires a deep knowledge of the various biodegradation processes occurring in different natural environments [13,14,15]. In this regard, over the last few years, polyesters such as polyhydroxyalkanoates, polylactic acid, poly(ε-caprolactone), poly(butylene succinate) and its copolymer poly(butylene succinate-*co*-adipate) have become the most promising candidates due to their sensitivity to enzymatic degradation [16,17]. Poly(butylene succinate-*co*-adipate) (PBSA) is a thermoplastic aliphatic polyester produced via a synthetic route from 1,4-butanediol, succinic acid, and adipic acid. However, the biosynthesis of these monomers from sugars allowed us to obtain fully bio-based PBSA [18,19]. Bio-derived PBSA has now found application in various industrial sectors (packaging and textile) with recent emerging trends in the agriculture field where PBSA is particularly employed in mulching films, bags, and plant pots [20,21], and in the automotive sectors as a composite material for internal building parts [22]. PBSA has good mechanical and thermal properties combined with excellent processability through conventional techniques such as extrusion, injection molding, and thermoforming [23,24]. Due to their low crystallinity and flexible polymer chains, both PBS and PBSA easily degrade in fresh water, seawater, soil burial, activated sludge, compost, and lipase solution with different rates depending on the comonomer ratios, molecular weight, branching, and specific environmental conditions such as pH, salinity, temperature, and crystallinity [25,26,27]. Interestingly, Fujimaki et al. [27] showed that the adipate copolymer PBSA had higher degradation rates than PBS in all different environments. It was demonstrated that microbial degradation begins with the adhesion of the enzyme to the plastic surface and continues with enzymatic catalysis of hydrolytic cleavage, resulting in a reduction in the polymer chain length. Then, the remaining oligomers and monomers are assimilated by microorganisms and converted into biomass, water, and carbon dioxide in aerobic conditions [28]. It was shown that soil microorganisms like bacteria and fungi can efficiently metabolize the polymer mainly to water and carbon dioxide [29]. In particular, Zhao et al. [30] reported the isolation of a fungus from compost, *Aspergillus versicolor*, that can induce the hydrolytic degradation of PBSA. Chien et al. [21] selected two elite *Aspergillus* strains to aid PBSA degradability in soil at moderate temperatures. In addition, Nishioka et al. [31] found that bacteria widely present in the soil and compost cause hydrolytic cleavage of ester linkages. Finally, Puchalski et al. [32] showed that compost is the most favorable degradation environment for PBSA. Although several studies have been conducted on the biodegradation of PBSA in compost and soil, only a few studies have reported its behavior in natural marine environments. Nakayama et al. [33] investigated the biodegradation of PBSA films in seawater in Osaka Bay at a depth of 1.5 m, and showed weight losses after 6 weeks in the range from 20 to 80%, depending strongly on factors such as seawater temperature, salinity, and microorganism population. Recently, Phosri et al. [34] showed that PBS blown films can degrade in about 100 weeks under seawater sand due to hydrolytic mechanisms and bioactive consumption.

The main disadvantage of PBSA is its high cost, which still limits its widespread distribution on a global market level. A viable strategy to increase its use in commodities consists of the development of PBSA-based composites containing low-cost biodegradable and renewable natural fibers as agricultural waste residues [35]. For this reason, various natural fibers have recently been considered as fillers for PBSA. For instance, hemp, feather, jute, flax, and cereal by-products such as wheat bran, straw, and rice husk have been used to produce inexpensive biodegradable biocomposites with enhanced properties [36]. In this context, bran is an abundant agricultural residue, being the main by-product of flour milling. It contains cellulose (~21%), lignin (~5%), and hygroscopic hemicellulose (~26%), starches, phenolic compounds, soluble and insoluble dietary fibers, and proteins. [37]. Recently, bran has been employed in PBSA composites to control plasticizer migration and the mechanical performances of PBSA blends [38,39]. It was demonstrated that the inclusion of hydrophilic fractions of wood-based fibers can induce the formation of micro-channels and cracks within the hydrophobic polymeric matrix, which improve water permeation, thus increasing the polymer fragmentation and biodegradation capacity in aqueous environments [40]. However, these studies all focused on the use of natural fibers in the PBS matrix rather than PBSA [41]. In particular, Sasimowski et al. [42] showed a significant effect of wheat bran content on the degradation kinetics of PBS-based composites using an accelerated aging chamber under controlled irradiation, water, and moisture conditions. They concluded that the fastest degradation occurred in bran, which is the preferred environment for microorganisms. To the best of our knowledge, PBSA/bran biodegradation behavior and material properties in natural seawater have not been investigated yet.

The current research work focused on the potential use of bran as a filler for PBSA to reduce the cost of the final product, preserving its processability and mechanical properties, and improving its biodegradation in seawater by exploiting bran’s high hygroscopicity. PBSA composites with different wheat bran content (5, 10, 15, and 20 wt.%) were produced by melt extrusion and processed by injection molding. The composites were morphologically and chemically analyzed by scanning electron microscopy (SEM) and Fourier transform infrared (FTIR), respectively. The thermal properties were studied by thermal gravimetric analysis (TGA) and differential scanning calorimetry (DSC). To investigate the effect of bran on the melt viscosity and processability, a rheological analysis of the composites was also carried out. The mechanical properties were determined by tensile tests conducted on dog-bone specimens. Finally, a biodegradation test on dog-bones of neat PBSA and PBSA/bran composite containing the optimized bran content was conducted in a seawater tank, monitoring the pH, temperature, oxygen content, and salinity to assess the effect of the filler on the PBSA biodegradation rate over time.

## 2. Materials and Methods

### 2.1. Materials

Poly(butylene succinate-*co*-adipate) (PBSA), commercially available as BioPBS™ FD92PM, was purchased from MCCP Germany GmbH (Mitsubishi Chemical Co., Tokyo, Japan) in the form of pellets. FD92PM is produced by the copolymerization of bio-based 1,4-butanediol, succinic acid, and adipic acid with the following characteristics: density 1.24 g/cm^3^ (23 ± 0.5 °C) [43]; melting temperature 84 °C; melt flow rate (MFR) 4 g/10 min (ISO 1133 190 °C/2.16 kg) [44]; butylene adipate content about 20 wt.% [45]. PBSA FD92PM is certified industrial/home compostable and biodegradable in soil by TÜV Austria and of food contact grade by EU10/2011 [46]. Wheat bran (B), used as a filler and biodegradation enhancer, was supplied from Barilla Spa (Parma, Italy) with particle sizes between 0.1 and 0.3 mm.

### 2.2. Composite Production

Bran particles and PBSA pellets were initially dried in an oven at 50 °C for about 24 h in a common atmosphere. Then, dried pellets of PBSA with 5, 10, 15, and 20 wt.% B (indicated as B5, B10, B15, and B20, respectively) were processed by a single-screw Brabender Extruder GmbH & Co. KG (Duisburg, Germany), with a feed mass rate of 15 kg/h and a nozzle size of 1.5 mm. The temperature profile adopted for all of the composites was 145/150/165/160/155 °C from the feed to the head zone. The screw rate was kept constant at 60 rpm (mean torque value of 40 Nm). The extruded filaments were cooled in a water bath at room temperature and reduced in pellets by an automatic cutter (Procut 3D Chinchio Sergio srl). Pellets were finally dried for 24 h in an oven at 50 °C and sealed in vacuum bags to avoid moisture capture before being subjected to the different characterization analyses.

### 2.3. Thermal Characterization

Thermogravimetric analysis (TGA) and derivative thermogravimetric analysis (DTGA) were performed with an STA 2500 Regulus Netzsch (NETZSCH-Gerätebau GmbH, Selb, Germany). About 10 mg of the sample was loaded into a ceramic pan and heated from room temperature to 600 °C at 10 °C/min under a nitrogen flow (20 mL/min). TGA was used to evaluate the thermal stability of bran and the effect of filler on the thermal stability of PBSA in the composites.

Differential scanning calorimetry (DSC) was performed by a Perkin Elmer DSC 6000 (Perkin Elmer Instrument, Waltham, MA, USA). About 15 mg of pellets was placed in an aluminum pan and subjected to a first heating from −60 °C to 120 °C (to remove any thermal history from processing), followed by cooling from 120 °C to −60 °C and a second heating to 120 °C under a nitrogen flow (20 mL/min at 10/20/10 °C/min, respectively). The melting temperature (*T*_m_) and the melting enthalpy (Δ*H*_m_) were determined by the second heating DSC curves. The crystallinity percentage, *X*_c_, in the PBSA/B composites was calculated as follows:

(1) $$X_{c}\left(\%\right)=\frac{{∆H}_{m}}{{∆H}_{m}^{0}\;\cdot \;(1-w_{f})}\;\cdot 100$$

where Δ*H*_m_ is the melting enthalpy of the sample; ${∆H}_{m}^{0}$ is the melting enthalpy of the 100% crystalline PBSA (142 J/g) [47]; and the *w*_f_ is the weight fraction of bran in the composite.

### 2.4. Rheological Analysis

The effect of bran on the viscosity of the composites was evaluated at 145 °C by an MCR 92 Rheometer (Anton Paar, Graz, Austria) using a plate–plate geometry with a 25 mm diameter and 1 mm gap. An amplitude sweep was used to select the optimal operating parameters to find the linear viscoelastic limit. Tests were run with oscillatory frequency sweeps from 0.05 to 100 Hz (0.314 to 628 rad/s) using a fixed strain of 0.2%. The complex viscosity η*, the loss modulus *G″*, and the storage modulus *G′* were all measured as functions of angular frequency ω. Furthermore, the flow behavior of virgin PBSA and PBSA/B composites was carried out by melt flow rate (MFR) measurements according to ASTM D1238 [48]. About 4 g of pellets were heated at 150 °C in the barrel and extruded through the normalized diameter (2.095 mm) under a constant load of 2.16 kg. Three replicates were performed for each sample, and the standard deviations for each data point were reported.

### 2.5. Fourier Transform Infrared Analysis

Fourier transform infrared (FTIR) analysis was carried out on PBSA, bran, and PBSA/B pellets, dried at 40 °C for about 24 h to remove moisture. FTIR spectroscopy was used to evaluate the possible intermolecular interactions between the functional groups of PBSA and bran by monitoring the shifts of the typical absorption peaks of the two components in specific regions. The FTIR spectra were recorded using a Perkin Elmer Spectrum One FTIR Spectrometer (Perkin Elmer, Waltham, MA, USA) with a Perkin Elmer Universal ATR Sampling Accessory in the wavenumber range of 4000 to 650 cm^−1^ at a 4 cm^−1^ scanning resolution.

### 2.6. Scanning Electron Microscopy and Stereo Microscopy

Scanning electron microscopy (SEM) was carried out to evaluate the dispersion of bran in the PBSA matrix using a COXEM Co. Ltd. Model EM-30N (Daejo, Republic of Korea). The PBSA and PBSA/B composite pellets were fractured in liquid nitrogen and the fractured surfaces were coated with a thin (5–6 nm) gold layer through an Edwards S150B Sputter Coater (Manor Royal, Crawley, West Sussex, UK). To complement the SEM analysis, optical microscopy was also carried out on the PBSA/B composite pellets using a Leica S9i stereo microscope (Wetzlar, Germany) equipped with a CCD camera.

### 2.7. Tensile Test

Tensile tests were conducted on PBSA and PBSA/B dog-bone specimens formed by a mini-injection press (ZWP Proma, Tychy, Poland). Neat PBSA and PBSA/B pellets were loaded within a thermostatic barrel of the injection press at 140 °C and after 1 min, the resultant melt was mechanically injected in the stainless-steel dog-bone mold and kept at 60 °C for another extra minute before removing the sample. The dog-bone specimens had the following sizes: length = 80 mm, big section width = 12 mm, 17 width = 4 mm, thickness = 2 mm. An Eden Prairie, MN, USA, MTS 50 KN system machine, outfitted with a 2 kN load cell and connected to a PC running the dedicated MTS Elite Software TW was used to conduct stress–strain testing at room temperature at a crosshead speed of 10 mm/min. Each tensile test was performed on five specimens of each sample and the mean values and relative standard deviations were reported.

### 2.8. Biodegradability Test in Seawater

**Figure 1:**
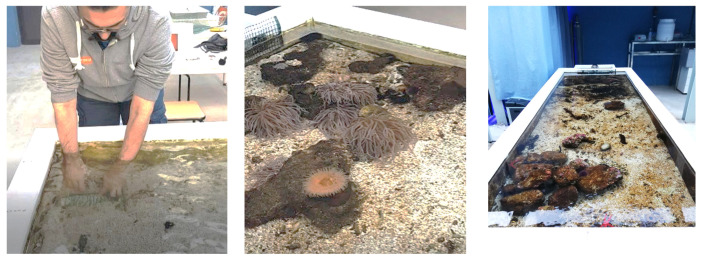
Seawater tank used for the biodegradation test (lasted 34 months).

A biodegradation test in seawater was conducted on PBSA and B15 dog-bone specimens at the Acquario of Livorno (Italy) to evaluate the effect of bran on the PBSA degradation rate. Each specimen was placed at a depth of about 4 cm under natural calcareous sand contained in a tank continuously fed with fresh seawater (Figure 1), which was taken at a 2 m depth and 50 m from the coast, filtered, and UV sterilized. The thickness of the sediment was about 6 cm, and gradually colonized with microorganisms and benthic creatures (e.g., ophire polychaetes and amphipods) that degrade the food residues given to the animals that inhabit the tank such as sea urchins, anemones, sea stars, mollusks, and crustaceans (Figure 1). As the tank is in communication with the sea by a continuous supply of outside seawater, the parameters of pH, oxygen, and salinity were practically identical to those of the external environment. Conversely, the seawater tank temperature was mainly affected by the internal room temperature. During the test, which lasted 34 months, these parameters were monitored daily. Temperature fluctuations were recorded across the whole biodegradation test. The oxygen was naturally developed by the algae and exchanged through the external environment; therefore, it was not necessary to add it. Unlike the temperature, the other parameters remained almost constant during the test and the average values of pH, oxygen, and salinity were 7.8, 4.1 g/L, and 37.4 g/L, respectively. The specimens were placed in the tank after weighing; every three months, three specimens of each sample were removed, and any adhering material and superficial moisture were extracted with absorbent paper, dried in an oven at 105 °C for 8 h, and then weighed. The last two samplings were conducted after 8 months from the last one. The weight losses, with respect to the initial weight of the samples, were evaluated and the mean values were reported. Changes in the mechanical properties were also monitored over time by carrying out tensile tests on specimens removed at each sampling. The mean values and relative standard deviations were reported.

## 3. Results and Discussion

### 3.1. Thermal Analysis

**Figure 2:**
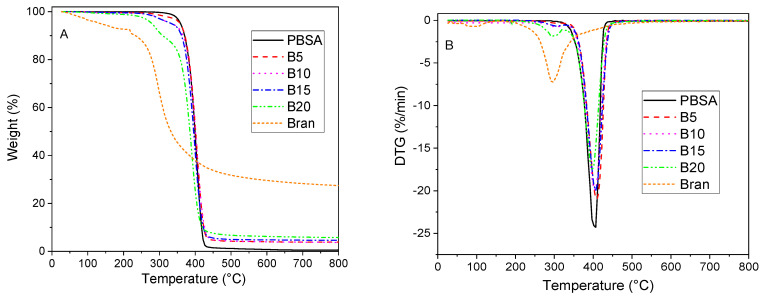
(**A**) TG and (**B**) DTG curves of bran, virgin PBSA, and PBSA/B composites (B5, B10, B15, and B20) under nitrogen atmosphere at a heating rate of 10 °C/min.

The TG and DTG curves of the bran, virgin PBSA, and PBSA/B composites are reported in Figure 2. As explained by Hejna et al., the bran showed a first weight loss of 10% at about 100 °C, corresponding to the moisture adsorbed due to its hygroscopicity [49]. Thermal decomposition started above 200 °C with a maximum peak at about 300 °C, indicating a good thermal stability in the temperature range typically adopted for processing PBSA via hot melt extrusion. The residue (30%) at 800 °C is attributable to the resultant char derived from cellulose, lignin, proteins, and starch present in the bran and the inorganic fraction [50]. PBSA started the thermal decomposition at 300 °C with a peak at about 400 °C and a negligible residue (0.5%) at 800 °C was observed, which agrees with the results of Seggiani et al. [51]. The PBSA/B composites exhibited two degradation steps: the first step between 200 and 350 °C is attributable to the bran degradation and the second one between 350 and 450 °C corresponds to the PBSA degradation. The residues at 800 °C are attributable to the resultant char and inorganic fraction of the bran.

**Figure 3:**
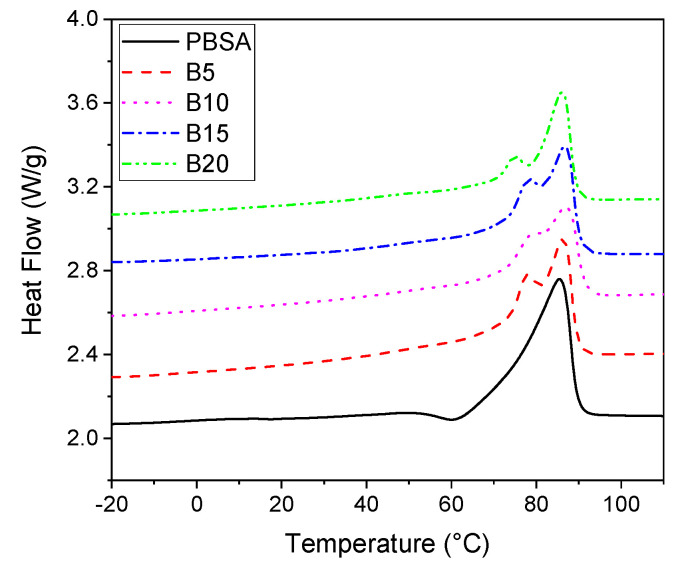
DSC thermograms of the PBSA and PBSA/B composites during the second heating scan at 10 °C/min.

Figure 3 shows the DSC thermograms of the second heating run of virgin PBSA and the PBSA/B composites. The melting temperature (*T*_m_), the melting enthalpy (Δ*H*_m_), and the crystallinity degree (*X*_c_) are reported in Table 1. The thermogram of the virgin PBSA showed a single endothermic peak at 85 °C and a small exothermic re-crystallization peak at about 60 °C. The presence of the exothermic peak at temperatures just before the melting event may be due to the rearrangement of crystals until their total melting [52]. The addition of bran to the PBSA matrix led to the presence of two melting peaks: a first shoulder peak (*T*_m1_) in the range 75–78 °C, followed by a second main peak (*T*_m2_) that matches the melting temperature of the virgin PBSA (85 °C). The addition of bran filler did not significantly affect the main melting temperature, but led to the formation of two crystalline phases with two distinct melting temperatures. This outcome is in agreement with other calorimetric studies conducted on PBS and PBSA that reported changes in the melting behavior of these semi-crystalline polymers as a result of filler addition [53]. All the composites showed a lower normalized melting enthalpy compared to the neat PBSA value, which resulted in a lower crystallinity degree of PBSA in the composites compared to the virgin PBSA. Thus, bran hinders PBSA polymer crystallization; a less perfect arrangement of the PBSA polymer crystal structures was achieved once the filler was dispersed into the matrix [42]. This phenomenon may also be associated with a slight reduction in the crystal size and a restricted polymer chain mobility in the presence of bran [54]. The reduction in the PBSA crystallinity is an important factor that can affect PBSA degradation as both PBSA hydrolysis and enzymatic degradation occurred first on the amorphous fractions, which were then followed by crystalline ones [32].

### 3.2. Rheological Analysis

**Figure 4:**
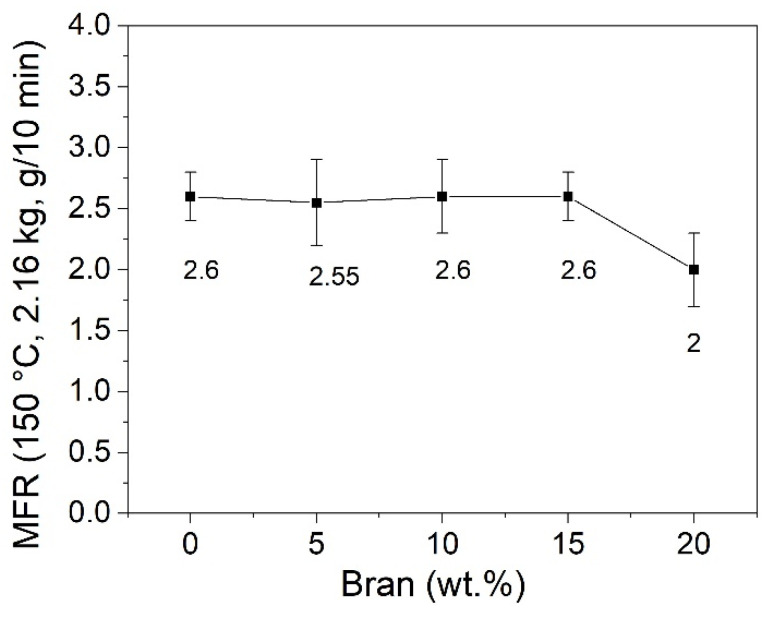
MFR values obtained at 150 °C for the virgin PBSA and PBSA/B composites (B5, B10, B15, and B20). Mean values ± standard deviations of three replicates.

**Figure 5:**
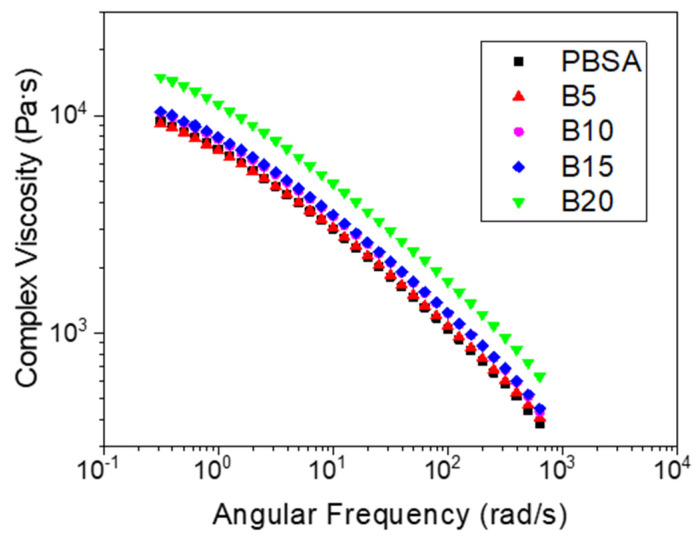
Complex viscosity versus the angular frequency of the PBSA and PBSA/B composites (B5, B10, B15, and B20) at 145 °C.

The melt fluidity measurements of the virgin PBSA and PBSA/B composites are reported in Figure 4 in terms of the MFR values obtained at 150 °C. As shown, the addition of bran up to 15 wt.% had a small effect on the MFR, remaining almost constant and equal to the MFR value of neat PBSA (about 2.6 g/10 min) while at 20 wt.% of bran, the MFR decreased to about 2 g/10 min, showing an increase in the composite melt strength. This change in the melt fluidity at 20 wt.% was confirmed by the complex viscosity η* measurements at 145 °C reported in Figure 5. The PBSA, B5, B10, and B15 curves overlapped approximately following the typical trend of the shear-thinning viscoelastic polymer material. An increase in η* was then observed as the bran content passed from 15 to 20 wt.%. This change in the melt viscosity may be caused by the friction between bran particles compared to the lower polymer–filler and polymer–polymer interactions. When the bran content became higher than 15 wt.%, the polymeric matrix became oversaturated with bran and the bran particles came into contact with each other, causing an increase in η* in accordance with the observed reduction in the MFR [55].

**Figure 6:**
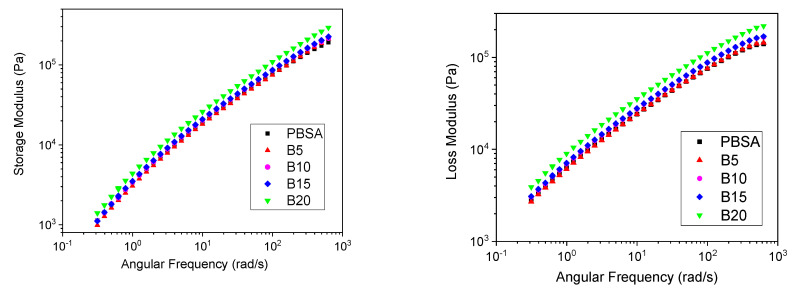
Storage (*G′*) and loss (*G″*) moduli versus angular frequency for the PBSA and PBSA/B composites (B5, B10, B15, and B20) at 145 °C.

**Figure 7:**
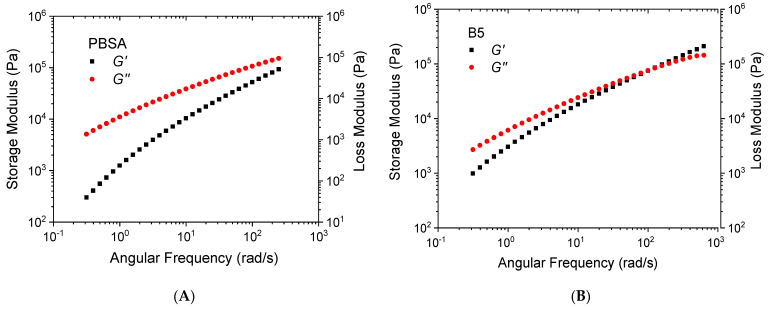

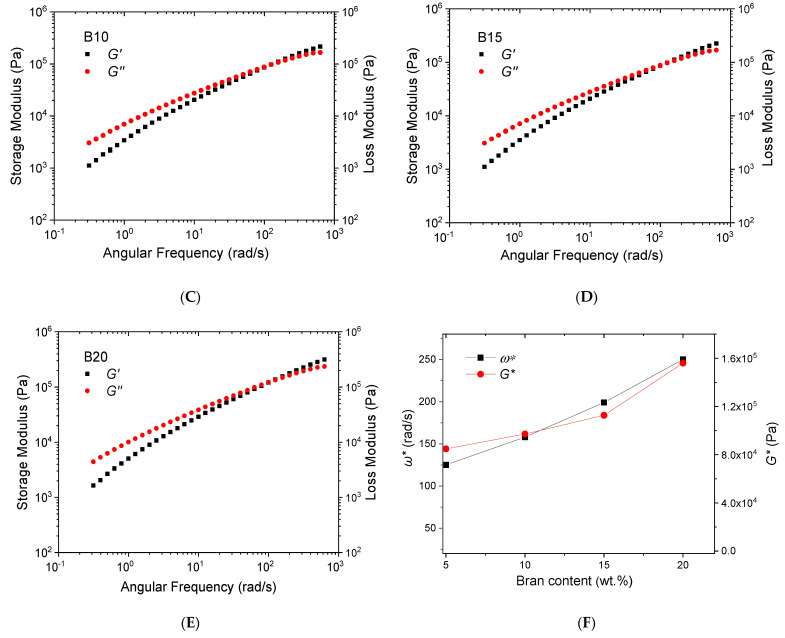
(**A**–**E**) Storage (*G′*) and loss (*G″*) modulus versus angular frequency of the PBSA and PBSA/B composites at 145 °C; (**F**) *G_cross_* and *ω_cross_* of the composites vs. bran content.

As reported in Figure 6, the increase in η* from B15 to B20 was determined by a growth in both the storage (*G′*) and loss (*G″*) moduli, which conferred the PBSA/B composite with improved resistance to flow. Figure 7A–E shows that for all the composites, the storage modulus was initially lower than the loss modulus, particularly in the low-frequency regions. As the frequency increased, *G′* increased with a steeper slope than *G″*, thus causing it to converge at a characteristic point known as the crossover. In general, the viscoelastic response of polymer composite systems is governed by filler/filler, filler/polymer, and polymer/polymer interactions. In this case, the increase in storage modulus can mainly be attributed to the bran particle–particle interactions that determine the achievement of a prevalent elastically deformable composite network [56]. The elastic response where *G′* > *G″* mainly occurs at high frequencies where the particles adhere to each other more easily. In addition, Figure 7F shows the crossover modulus *G*_cross_ (average value between *G′* and *G″*) and the corresponding frequency *ω*_cross_ versus the bran content. A higher increase in *G*_cross_ and *ω*_cross_ was observed passing from B15 and B20, confirming the occurrence of bran saturation in the polymer matrix and consequent elastic particle–particle interactions dominant over viscous polymer–polymer and polymer–particle interactions. 

### 3.3. Fourier Transform Infrared Analysis

**Figure 8:**
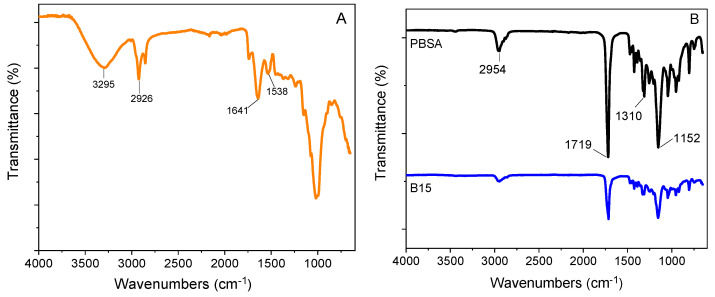
FTIR spectra of (**A**) bran, (**B**) neat PBSA, and the composite with 15 wt.% bran.

Figure 8A shows the FTIR spectra of the used bran sample with the typical sharp –OH group stretching visible in the 3600–3000 cm^−1^ region. The peak at 3295 cm^−1^ can be attributed to the intramolecular hydrogen bonds of cellulose II, whereas the small peak at 2926 cm^−1^ corresponded to the stretching vibrations of the C–H bonds in the hemicelluloses and cellulose [57]. Moreover, the bands at 1641 cm^−1^ and 1538 cm^−1^ were attributed to the proteins in the bran, specifically to the amide I and amide II vibrations, respectively. The bands near 1641 cm^−1^ were also associated with the C=O deformation of xylan, which is the main constituent of hemicellulose [58]. The FTIR spectra of the PBSA/B composites perfectly overlapped. As an example, Figure 8B shows the FTIR spectra of the neat PBSA and B15 composite. In the spectrum of PBSA, the peaks at 2954 cm^−1^ and 1310 cm^−1^ were attributed to the asymmetric and symmetric vibration of the CH_2_ groups, and at 1719 cm^−1^ to the C=O vibration, while the band at 1152 cm^−1^ was due to the –C–O–C– stretching of the PBSA ester groups [51]. The B15 composite spectrum showed no significant differences compared to that of PBSA, with the characteristic peaks overlapping each other. Moreover, no significant shift or new bands attributable to chemical interactions between the filler and polymer matrix were observed. In conclusion, the addition of bran only reduced the intensity of significant PBSA peaks, indicating that bran acts as an inert filler [59].

### 3.4. Morphological Analysis

**Figure 9:**
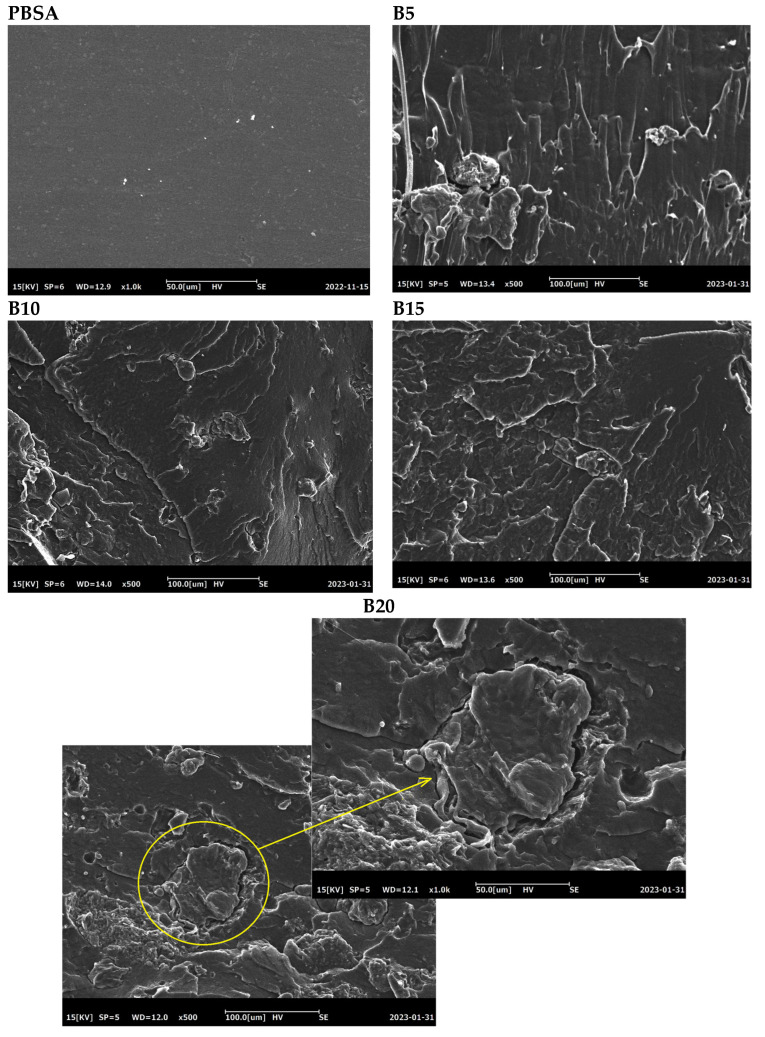
SEM images of bran and the freeze fractured surfaces of the PBSA and PBSA/B composite pellets. Poor particle/matrix adhesion is highlighted in the circle for the B20 sample.

SEM photographs of the bran and fractured surfaces of the neat PBSA and PBSA/B composites are shown in Figure 9. Wheat bran particulate consisted of flakes mainly in the 80–150 μm size range, but smaller size fractions were also observed. Indeed, the process of separating the bran from the seed core produced polydisperse particle fractions that differed in dimension, composition, and lignocellulosic material content [60]. No significant morphological differences were observed when comparing the B5, B10, and B15 micrographs where the bran flakes appeared homogeneously dispersed without any visible agglomeration, surface cracks, and holes. Despite the poor affinity between the hydrophilic bran and the hydrophobic PBSA, good filler/matrix adhesion and filler dispersion were observed for composites containing up to 15 wt.% bran. On the other hand, at 20 wt.%, the filler/matrix adhesion worsened, leading to detachment of the bran flakes from the matrix (Figure 9). In conclusion, the SEM images support the evidence that the 15 wt.% bran content represents the limit beyond which the bran cannot be uniformly dispersed within the polymeric matrix. 

**Figure 10:**
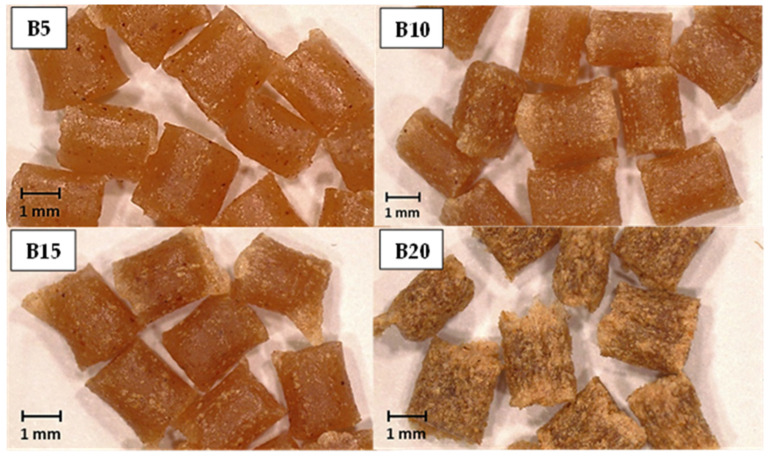
Stereo microscope images of the PBSA/B composite pellets.

Stereo microscope images of the PBSA/B composite pellets are reported in Figure 10. The increase in the bran content led to a change in the composite color, which resulted in being gradually darker with increasing filler content. No significant differences between the B5, B10, and B15 composites were observed: pellets appeared homogeneous with the bran flakes incorporated within the polymeric matrix. Conversely, B20 pellets presented more corrugated surfaces with bran fibers accumulating on the pellet exteriors, forming voids and asperities that indicate poor filler absorption and the achievement of a bran saturation point between 15 wt.% and 20 wt.%.

### 3.5. Mechanical Properties

Table 2 shows the tensile properties of the dog-bone specimens. Interestingly, the results showed that the various composite formulations maintained good mechanical properties below 15 wt.% compared to neat PBSA. Indeed, B5, B10, and B15 showed a yield strength, elongation at yield, and stress at break similar to the neat PBSA. The Young’s modulus instead gradually increased with the addition of bran from 0.29 GPa (PBSA) to 0.37 GPa (B15). According to the increase in stiffness, the elongation at break decreased with a filler addition from 400% (PBSA) to 207% (B15). This is in agreement with Sasimowski et al. [53], which demonstrated an increase in the hardness and stiffness with the bran content in the PBS composites. In summary, the addition of bran filler caused a change in the mechanical properties, even at low concentrations. However, the differences observed below 15 wt.% were not as relevant as those measured once this threshold was exceeded. A collapse in the mechanical properties was in fact registered when passing from 15 to 20 wt.% bran. Moreover, B20 showed no plasticity region with the yield strength and the elongation at yield matching the stress at break and the elongation at break, respectively. This behavior can be attributed to the presence of large bran flake agglomerates in the over-loaded matrix that act as defect points from which brittle crack propagation across the specimen can start [61]. This result is in agreement with the rheological, and morphological findings that showed a 15 wt.% bran content as the load threshold beyond which bran flakes make the composite stiff, brittle, and susceptible to fracture at low stress.

In conclusion, B15 resulted in being the optimal formulation as it maximized the natural filler content to be added to the PBSA matrix while preserving the mechanical properties of the resulting composite. Thus, B15 was selected for the biodegradation test in seawater. 

### 3.6. Biodegradation in Seawater

**Figure 11:**
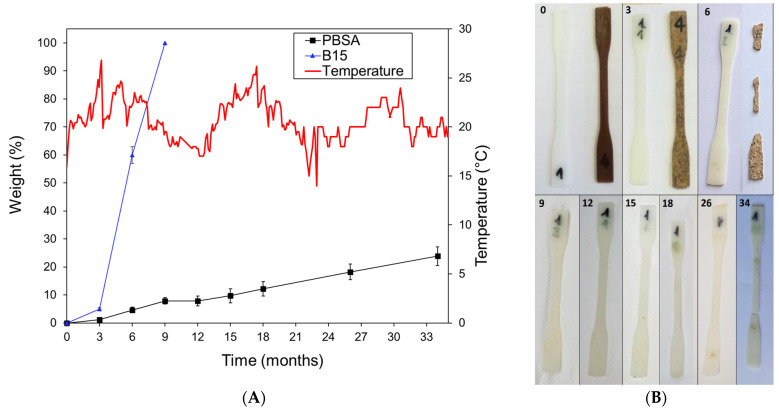
(**A**) The PBSA and B15 weight loss (%) over time during the biodegradation test in the seawater tank. Mean values ± standard deviation of 3 replicates. Average pH 7.8, O_2_ concentration 4.1 g/L, and salinity 37.4 g/L). (**B**) Starting dog-bone specimens and after the periodic samplings; the sampling month number is shown at the top.

Figure 11A reports the weight losses of the PBSA and B15 dog-bone specimens in seawater and the recorded seawater temperature over time. Minor temperature fluctuations of about 7–8 °C were measured between summer and winter. The results demonstrate a slow linear degradation of the neat PBSA that is independent of the seasonal temperature changes, showing a weight loss of about 25% after 34 months. This value was lower than Nakayama et al. [33], who obtained a similar weight loss after only a few months, but worked with thin PBSA films (100 µm) having a much higher surface area exposed to the aqueous environment than our dog-bone samples. On the other hand, the composite with the 15 wt.% bran showed a weight loss of about 58% after 6 months, degrading completely within 9 months (Figure 11A,B). The hydrophilicity of the bran may have caused the flakes to swell in the aqueous medium, facilitating the fragmentation of the specimen with a consequent increase in the surface area exposed to microbial attack. These results confirm the positive effect of bran on the degradation of PBSA as reported by Sasimowski et al. [43], who showed that after 70 days of composting, the weight of the samples made from neat PBS was only reduced by 4.5%, but samples with 10 wt.% bran decreased by 15.1%.

Table 3 shows the results of the tensile tests carried out on the dog-bone specimens after each sampling. As expected, the tensile tests showed a gradual change in the mechanical parameters for the neat PBSA, while for the B15 composite, a marked loss in mechanical performance was observed after 3 months in seawater, corresponding to a weight loss of about 5% (Figure 11A), and after 6 months, the specimens resulted in being fragmented and markedly degraded. Interestingly, the Young’s modulus, the yield strength, the elongation at yield, and the stress at break of the neat PBSA increased after 6–9 months of incubation under sediment in the seawater tank and then slowly decreased over time. This was probably caused by the crystallization of seawater salts within the PBSA porous specimen matrix after drying, which made the composite structure more rigid.

The observed effect of the bran addition on the weight loss and mechanical properties in seawater is consistent with a recent study on the degradation of PBS/bran composites conducted in soil and compost [42]. However, the current research work extends the study of the biodegradation of PBSA/bran composites to natural marine aqueous environments. Despite the current study not investigating the complex biological and enzymatic mechanisms involved in the biodegradation of both PBSA and bran, it showed that by suitably adding bran to the PBSA-based composites, it is possible to tune their mechanical properties and the rate of biodegradation in natural environments such as a marine one for possible applications in this field.

## 4. Conclusions

Composites of PBSA and wheat bran, a by-product of the flour milling, were successfully processed via extrusion and injection molding up to 20 wt.% bran. SEM investigation showed a uniform distribution of the bran flakes in the PBSA matrix and a good adhesion flake/matrix up to 15 wt.% bran. At 20 wt.% bran, aggregates of bran flakes and poor adhesion were observed. A gradual increase in stiffness and a decrease in the elongation at break were obtained with an increase in the bran content up to 15 wt.%, but at 20 wt.% bran, the mechanical performance was strongly reduced. The results clearly show that the loading of 15 wt.% bran represents a threshold beyond which bran flakes make the composite stiff, brittle, and susceptible to fracture at low stress.

The incubation of neat PBSA and B15 specimens under natural sandy sediment in a seawater tank showed a slow biodegradation rate for PBSA, reaching a weight loss of 25% after 34 months, while a complete biodegradation was observed for B15 after 9 months. The high biodegradation rate of B15 may be attributable to the hydrophilicity of bran. The swelling of bran flakes favors the fragmentation of the specimen with a consequent increase in the surface area of the polymeric matrix exposed to microbial attack. In conclusion, the use of wheat bran (0.1–0.3 mm) up to 15 wt.% as filler in PBSA-based composites represents an opportunity to valorize this abundant agro-food by-product, lowering the cost of the final PBSA-based products and maintaining the mechanical properties of the resulting composites at a satisfactory level to meet the design requirements of many everyday objects. In addition, by suitably adding bran to PBSA, it is possible to modulate the mechanical properties of the composite and the rate of biodegradation in natural environments such as a marine one for possible applications in this field.

## Figures and Tables

**Table 1:** The thermal parameters and crystallinity degree of the PBSA and PBSA/B composites. Δ*H*_m_* (J/g) values were normalized to the effective PBSA content.

Sample	*T*_m1_ (°C)	*T*_m2_ (°C)	Δ*H*_m_* (J/g)	*X*_c_ (%)
PBSA	-	85.5	45.6	32.1
B5	77.7	86.6	30.5	22.6
B10	78.7	87.8	30.4	23.8
B15	77.8	86.8	35.2	29.2
B20	74.7	86.2	24.9	21.9

**Table 2:** The tensile properties of the PBSA and PBSA/B composites. The mean value ± standard deviation of five replicates.

Sample	Young’s Modulus (GPa)	Yield Strength (MPa)	Elongation at Yield (%)	Stress at Break (MPa)	Elongation at Break (%)
PBSA	0.29 ± 0.05	17.50 ± 1.10	14.50 ± 0.30	21.10 ± 0.50	400.00 ± 53.00
B5	0.34 ± 0.01	17.60 ± 0.93	16.60 ± 2.32	23.70 ± 2.36	306.50 ± 14.27
B10	0.39 ± 0.03	18.41 ± 1.87	19.22 ± 2.91	21.95 ± 1.91	200.50 ± 30.07
B15	0.37 ± 0.06	17.09 ± 0.75	16.53 ± 1.49	21.08 ± 1.93	207.00 ± 14.74
B20	0.44 ± 0.02	*	*	16.67 ± 1.43	72.13 ± 22.24

(*) Yield strength and elongation at yield equaled the stress at break and elongation at break, respectively.

**Table 3:** The tensile properties of the PBSA and B15 composites during the biodegradation test in a seawater tank. Mean values ± standard deviation of 3 replicates.

Time (Months)	Young’s Modulus (GPa)		Yield Strength (MPa)		Elongation at Yield (%)		Stress at Break (MPa)		Elongation at Break (%)	
	PBSA	B15	PBSA	B15	PBSA	B15	PBSA	B15	PBSA	B15
0	0.29 ± 0.05	0.37 ± 0.06	17.50 ± 1.10	17.09 ± 0.75	14.50 ± 0.30	16.53 ± 1.49	21.00 ± 0.50	21.08 ± 1.93	400.00 ± 53.00	207.00 ± 14.74
3	0.20 ± 0.04	0.20 ± 0.09	20.52 ± 1.54	19.01 ± 0.75	22.11 ± 0.32	17.99 ± 0.75	29.10 ± 0.66	3.60 ± 0.30	260.20 ± 55.50	4.50 ± 0.30
6	0.24 ± 0.06	-	23.41 ± 1.98	-	22.32 ± 0.43	-	26.71 ± 0.73	-	210.25 ± 32.30	-
9	0.34 ± 0.07	-	18.20 ± 1.56	-	26.75 ± 1.22	-	20.88 ± 0.86	-	247.75 ± 31.30	-
12	0.17 ± 0.08	-	16.75 ± 2.04	-	20.11 ± 1.51	-	15.38 ± 0.93	-	30.15 ± 26.30	-
15	0.14 ± 0.09	-	18.61 ± 2.84	-	22.23 ± 2.01	-	16.46 ± 1.10	-	27.85 ± 5.30	-
18	0.19 ± 0.11	-	*	-	*	-	10.52 ± 1.20	-	7.90 ± 1.30	-
26	0.11 ± 0.12	-	*	-	*	-	9.01 ± 1.20	-	6.88 ± 1.20	-
34	-	-	-	-	-	-	-	-	-	-

(-) Not evaluable; (*) Yield strength and elongation at yield equal to stress at break and elongation at break, respectively.

## Data Availability

Raw data can be available upon request by contacting the corresponding author. All of the processed data are reported in the text.
